# The Mortality of Abdominal Section

**Published:** 1900-07-21

**Authors:** 


### THE MORTALITY OF ABDOMINAL SECTION.

There are few points of more importance in regard to the treatment of abdominal and pelvic diseases than a due appreciation of the risks which are inseparable from abdominal section, for in proportion as, under the influence of improving technique, we find these risks fading into nothingness, so does the field in which operative interference, either for treatment or diagnosis, may be recommended with a good conscience, enlarge enormously. From this point of view especially, as well as for many other reasons, great interest attaches to a recent paper by Dr. William Duncan[1] on a hundred consecutive cases of abdominal section without a death (since made up to 108 cases). He says that on October 7th, 1897, a woman on whom he had operated for extra-uterine gestation, at the Chelsea Hospital for Women, died suddenly on the seventh day from pulmonary embolism, when she appeared to be doing perfectly well. From that date up to June 10th, 1900, he has had 100 consecutive cases of abdominal section without a death; and, as a footnote to his paper, he states that since the latter date, there are eight more cases to be added to the list, making altogether 108 successful cases. This is a very remarkable and very interesting series, and it shows well the practical freedom from risk with which the abdominal cavity may be opened when full and proper precautions are taken to avoid septic infection. Among the cases included in the list are 17 hysterectomies and 3 myomectomies for uterine fibroids, 23 operations for ovarian cysts, 11 for extra-uterine gestation, 9 for double pyosalpinx, 7 for tubo-ovarian cysts (5 suppurating), 12 for diseased and adherent appendages, and 3 exploratory incisions. Now in regard to the precautions taken it is to be noted that opening out of the operating-room is a sterilising-room with boilers, &c., by means of which all instruments, towels, operating aprons, &c., are rendered completely aseptic, and also that Dr. Duncan says, "personally, I always prefer, if possible, that no other fingers than my own shall enter the peritoneal cavity." After being shaved and thoroughly scrubbed with carbolic soap the day before the operation, the patient's abdomen is covered with a layer of wool soaked in a 1 in 1,000 solution of perchloride of mercury, which is kept in position until the time of the operation. The operator and assistants scrub hands and arms with soap, and then soak them in 1 in 2,000 hot solution of perchloride of mercury. The instruments are always sterilised just before the operation, and tranferred direct from the steriliser into a tray of plain boiled water. The silk (nothing else is used) is thoroughly sterilised, and then kept ready for use in bottles full of carbolic solution. Ordinary sponges, thoroughly cleansed, are used. During the operation they are simply wrung out of hot sterilised water. If used in purulent cases, or where the contents of an ovarian cyst consist of dirty grumous material, they are destroyed. Flushing with plain sterilised water at a temperature of 110 deg. F. is used in all cases where a cyst with a purulent or unhealthy grumous contents bursts during the operation, and it is also employed after some operations on ruptured tubal pregnancies, when it is required as a stimulant. Drainage is now hardly ever used, unless there be oozing from torn extensive adhesions low down in the pelvis, which cannot be stopped by ligature or by a few minutes' application of pressure forceps. Every care is taken to control all oozing before closing the peritoneal cavity. If drainage has to be employed it takes the form of a piece of indiarubber tubing or iodoform gauze. It is interesting to observe how different is the technique among different operators in regard to some matters, and yet how all the most successful seem to hold to the most absolute cleanliness, the most careful removal of effusion (in other words, of foreign substances), and the most complete control of oozing—plain and well recognised surgical principles, applicable to other wounds besides those involving the peritoneum.

[1] Lancet, July 7.

